# Validating distribution models for twelve endemic bird species of tropical dry forest in western Mexico

**DOI:** 10.1002/ece3.3160

**Published:** 2017-08-19

**Authors:** Miguel A. Ortega‐Huerta, Jorge H. Vega‐Rivera

**Affiliations:** ^1^ Estación de Biología Chamela Instituto de Biología Universidad Nacional Autónoma de México Chamela Jalisco México

**Keywords:** actual distribution model, field occurrence data, prediction threshold

## Abstract

Considering the high biodiversity and conservation concerns of the tropical dry forest, this study aim is to predict and evaluate the potential and current distributions of twelve species of endemic birds which distribute along the western slope of Mexico. The main goal is to evaluate altogether different methods for predicting actual species distribution models (ADMs) of the twelve species including the identification of key environmental potential limiting factors. ADMs for twelve endemic Mexican birds were generated and validated by means of applying: (1) three widely used species niche modeling approaches (ENFA, Garp, and Maxent); (2) two thresholding methods, based on ROC curves and Kappa Index, for transforming continuous models to presence/absence (binary) models; (3) documented habitat–species associations for reducing species potential distribution models (PDMs); and (4) field occurrence data for validating final ADMs. Binary PDMs' predicted areas seemed overestimated, while ADMs looked drastically reduced and fragmented because of the approach taken for eliminating those predicted areas which were documented as unsuitable habitat types for individual species. Results indicated that both thresholding methods generated similar threshold values for species modeled by each of the three species distribution modeling algorithms (SDMAs). A Wilcoxon signed‐rank test, however, showed that Kappa values were generally higher than ROC curve for species modeled by ENFA and Maxent, while for Garp models there were no significant differences. Prediction success (e.g., true presences percentage) obtained from field occurrence data revealed a range of 50%–82% among the 12 species. The three modeling approaches applied enabled to test the application of two thresholding methods for transforming continuous to binary (presence/absence) models. The use of documented habitat preferences resulted in drastic reductions and fragmentation of PDMs. However, ADMs predictive success rate, tested using field species occurrence data, varied between 50 and 82%.

## 1 INTRODUCTION

### 1.1 Statement of the problem

It has become a common practice to generate species distribution models (SDMs) based on the use of occurrence records obtained from electronic museum databases. However, because of the historical nature of the species' presence data, there is a lack of correspondence between the wide span of time in which species occurrence data were collected and the date and conditions of key spatial (mapped) environmental variables, such as the land cover type. This disparity is particularly problematic in places with high rates of land use/land cover changes. This study's main challenge is to evaluate how realistic are SDMs in replicating the likelihood for recording species on the ground (e.g., Austin, 2007), which entails the achievement of two interrelated objectives: (1) modeling the actual distribution of 12 Mexican endemic species of birds and (2) validating models' performance using field occurrence data. The applied approach consisted in evaluating the performance of a group of three of the most used algorithms to generate SDMs (Garp, Maxent, and ENFA), with less emphasis on comparing between algorithms; a main assumption is that no single modeling algorithms conveys all answers for modeling specie distributions (e.g., Qiao, Soberón, & Peterson, 2015).

This study undertakes main species distribution modeling challenges by proposing concrete and practical solutions; a) transforming continuous to binary (presence/absence) SDMs, b) identifying potential distributional limiting factors, c) obtaining SDMs which represent the most likely areas actually occupied by the species, and d) validating these later SDMs using species occurrence data sampled on the field.

### 1.2 Study Species: Endemic species of the dry forest of western México

Considering the high levels of biodiversity and endemism found in Mexico's western slope (Escalante‐Pliego, Navarro‐Sigüenza, & Peterson, 1998; Peterson & Navarro, 1999, 2000) and the region's conservation concerns (e.g., Portillo‐Quintero & Sánchez‐Azofeifa, 2010), a main objective of this study is to predict and evaluate the potential and current distributions of twelve species of endemic birds which distribute along the tropical dry forest on the western slope of Mexico. These bird species are considered important community components, and they tend to show specialized habitat usage, also showing proportionally fastest population declines than other species with wider distribution patterns (Stotz, Fitzpatrick, Parker, & Moskovits, 1996).

Among the Mexican endemic bird species that we have recorded on the field across the country's western Pacific slope, twelve species were selected (Table 1) because their occurrence represents the array of conditions found in the tropical dry forest. These 12 species are strongly associated with the tropical dry forest and environments with a matrix dominated by this vegetation formation; they can occur in open or partially open areas (e.g., *Passerina leclancherii*), forest edges and scrub land (e.g., *Thryophilus sinaloa*), or in rural towns and dirt roads (e.g., *Ortalis poliocephala*,* Cacicus melanicterus*,* Trogon citreolus*).

**Table 1:** Number of species presence records with different geographic location (museum species records) and species occurrence records sampled on the field

Species	Museum species records	Field species occurrence sites (1 × 1 km cell)
*Cacicus melanicterus*	135	62
*Chlorostilbon auriceps*	61	23
*Deltarhynchus flammulatus*	26	26
*Granatellus venustus*	48	29
*Melanerpes chrysogenys*	196	87
*Ortalis poliocephala*	41	58
*Passerina leclancherii*	117	78
*Polioptila nigriceps*	38	12
*Pheugopedius felix*	123	92
*Thryophilus sinaloa*	151	92
*Trogon citreolus*	146	70
*Vireo hypochryseus*	106	62

**Figure 1:**
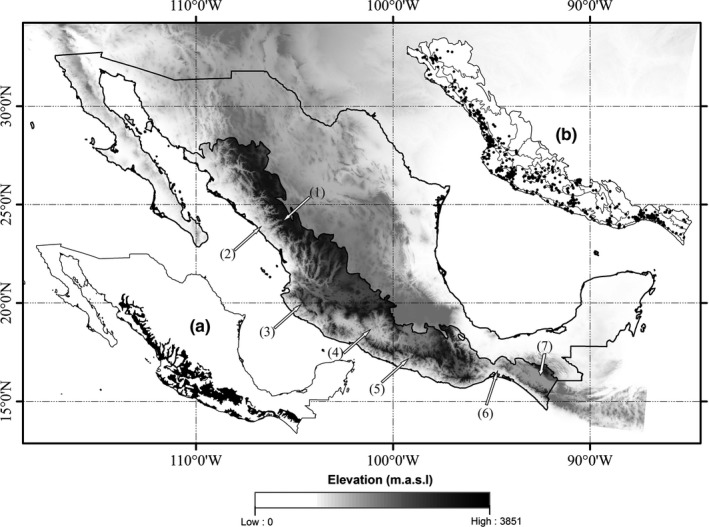
Study region. The central map of Mexico shows the study area delimitation (darker tones on the digital elevation model) formed by adding 37 physiographic provinces which include the original distribution of the tropical dry forest (Rzedowski, 1978) along the Pacific slope (inset a). Inset b shows the distribution of the 1189 species' occurrence point data within the physiographic provinces, corresponding to 12 species of endemic birds. The arrows point out physiographic provinces mentioned in the text: (1) Sierra Madre Occidental, (2) Pie de la Sierra, (3) Sierras de Jalisco y Colima, (4) Depresión del Balsas, (5) Cordillera Costera del Sur, (6) Itsmo de Tehuantepec, (7) Depresión Central de Chiapas

Notwithstanding that these species' general geographic pattern follows the tropical dry forest along the Pacific coast, from southern Sonora state to Chiapas (see Figure 1), particular patterns vary among the 12 selected species; species introduce through central Mexico into the Balsas Depression (e.g., *Cacicus melanicterus*) or continue their distribution to Chiapas (*Chlorostilbon auriceps*,* Melanerpes chrysogenys*,* Pheugopedius felix*,* Vireo hypochryseus*). Other species (*Deltarhynchus flammulatus*,* Passerina leclancherii*,* Trogon citreolus*) end their distribution in southern Oaxaca state, avoiding the wettest areas of the Tehuantepec Isthmus which acts as a physical barrier. Species such as *Thryophilus sinaloa*,* Pheugopedius felix*,* Vireo hypochryseus*,* Polioptila nigriceps,* and *Cacicus melanicterus* reach their northern distribution in southern Sonora, while other species (*Deltarhynchus flammulatus*) avoid the lowlands of northern Sinaloa, where the tropical dry forest separates from the coast and penetrates into canyons and mountains of the Sierra Madre Occidental. This later pattern results in the highest sites where the tropical dry forest distributes in Mexico (between 1,500 and 1,800 m.a.s.l).

### 1.3 Species distribution models

The modeling of species distribution has been also referred as modeling the “ecological niche,” “habitat suitability,” and “potential distribution” approaches aimed to identify species distribution areas and with this the limiting factors determining species distribution patterns (e.g., Elith & Graham, 2009; Soberon & Nakamura, 2009). These methods are conceptually related, and they have the same correlative nature: Field observations of species occurrences are related to environmental factors by means of a wide number of mathematical and statistical algorithms (Qiao et al., 2015). The application of these methods for generating models describing those places suitable for the distribution of species is generally referred as species distribution models (SDMs) (Elith & Graham, 2009; Elith et al., 2006; Franklin, 2010a, 2010b; Guisan & Thuiller, 2005; Loiselle et al., 2003). Some authors, however, make the distinction between niche and distribution or habitat models: While the former convey information about a species' environmental preferences, the latter just means its geographic projection without any other spatial or time projection (Owens et al., 2013). Habitat suitability models can be considered as operational applications of the ecological niche modeling which is based on a set of environmental variables predicting a species presence/absence (Hirzel & Le Lay, 2008).

### 1.4 Potential vs actual SDMs

This study applies the term “species distribution model” because it describes both the modeling approach and the resulting outcome (Franklin, 2010a, 2010b). By focusing on SDMs, we avoided the unresolved debate about what version of a species' ecological niche (fundamental vs realized) the SDMs represent (e.g., Araújo & Guisan, 2006; Elith & Leathwick, 2009). However, we made a key distinction between two terms usually related to the dichotomy between fundamental vs realized niches: species potential distribution models (PDMs) and species actual distribution models (ADMs). PDMs are generated by using abiotic environmental (bioclimatic and topographic) variables as predictors (e.g., Franklin, 2010b), while ADMs were derived from PDMs but excluding areas of unsuitable habitats on a species by species basis. Our PDMs are equivalent to those areas defined by Peterson and Soberón (2012) that have been accessible to the species over a relevant time period, containing the abiotic conditions required for a species' survival and growth, while the ADMs represent the species' most likely occupied areas.

### 1.5 Evaluation and selection of species distribution models

The development and variety of species distribution modeling algorithms (SDMAs) is revealed by the amount of scientific papers (see Guisan et al., 2013) and books (Costa, 2009; Drew, Wiersma, & Huettmann, 2011; Franklin, 2010b; Peterson et al., 2011; Scott, Raven, Heglund, & Morrison, 2002) published during the last decade. SDMAs differentiate among each other because of the way model's distribution response is obtained, how prediction variables are selected and relevant variables are identified and weighted, how the fitted functions are defined, the degree of interaction between variables and how prediction is performed onto geographic space (Elith et al., 2006). Correlative SDMs can also be classified whether they use species presence/absence or only‐presence data (Tsoar, Allouche, Steinitz, Rotem, & Kadmon, 2007).

Considering the variety of SDMAs and the expectations for applying their analyses and results to a wide range of fields (Araújo & Peterson, 2012), model evaluation is considered one of the most challenging and important steps in applying the ecological niche modeling or species distribution modeling (SDM) (e.g., Rodríguez‐Rey, Jiménez‐Valverde, & Acevedo, 2013). Model evaluation is a necessary modeling step because of the high differences found among the outcomes obtained from applying different SDMAs (Marmion, Parviainen, Luoto, Heikkinen, & Thuiller, 2009).

Previous work on evaluating distribution models for bird species, include the comparison of different SDMAs: discriminant, logistic regression, and neural networks (Manel, Dias, Buckton, & Ormerod, 1999; Manel, Dias, & Ormerod, 1999), Ecological Niche Factor Analysis (ENFA) and generalized linear model (GLM) (Brotons, Thuiller, Araújo, & Hirzel, 2004; Hirzel, Helfer, & Metral, 2001), and Bioclim, Domain, Garp, and MaxEnt (Hernandez, Graham, Master, & Albert, 2006). Evaluating SDMs' predictive performance and statistical significance requires two independent sets of species presence/absence data: one for model calibration and other for model evaluation (Peterson et al., 2011). Elith et al. (2006) elaborated what they considered the most comprehensive study to evaluate the application of 16 modeling methods for comparing the modeling of 226 species using independent datasets. The use of field data to validate SDMs is less common: Tsoar et al. (2007) evaluated several SDMAs' performance for modeling snails, birds, and bats in Israel; West, Kumar, Brown, Stohlgren, and Bromberg (2016) tested a Maxent model for an invasive grass in the Rocky Mountain National Park using independent presence/absence data collected during 6 years; Rebelo and Jones (2010) ground validated ENFA and Maxent distribution models for a Portugal's rare bat species, using acoustic transects. In this study, three of the most used SDMAs are applied and their performance is tested in modeling the potential and actual distribution of 12 endemic bird species in Mexico.

## 2 METHODS

### 2.1 Study area

The study area corresponds to Mexico's western slope. Its delineation consisted in adding the 37 physiographic provinces (Cervantes‐Zamora et al., 1990) that include the original distribution of the tropical dry forest as mapped by Rzedowski (1990) (Figure 1).

Mexico's richness of endemic bird species is remarkable; approximately 10% of the 1,050 bird species reported for the country are considered endemic, with the tropical dry forest of western Mexico being one of their most important habitats (e.g., Peterson & Navarro, 2000). Just within the Chamela‐Cuixmala Biosphere Reserve, which is only 130 km^2^ in size, there are 24 endemic species recorded, and from these 20 are endemic to western Mexico (Arizmendi, Berlanga, Márquez‐Val Delamar, Navarijo, & Ornelas, 1990; Vega Rivera, Arizmendi, & Morales‐Pérez, 2010). However, similarly to what other types of ecosystems across the country are facing, the tropical dry forest's shrinking and impoverishing have compromised the conservation of this ecosystem's biodiversity; by the 1990s, just 27% were tropical dry forest in good conservation conditions (Trejo & Dirzo, 2000). The tropical dry forest distributed on the Mexican western slope is considered among the most extensive and conserved areas across the Mesoamerican region and its regional and global biological importance is recognized (Ceballos et al., 2010).

### 2.2 Species occurrence data

Twelve endemic bird species were selected for this study (see Table 1) among 47 endemic species reported (Gordon & Ornelas, 2000; Vega Rivera et al., 2010), based on the availability of data in the form of scientific collections and our knowledge and field experience sampling birds within western Mexico's tropical dry forest.

The approach taken consisted in using museum records for generating (training and evaluation) the species' potential distribution models (PDMs). On the other hand, species occurrence data sampled on the field (FOD) were used for validating species actual distribution models (ADMs). The ADMs consisted of the reduced version of PDMs after excluding predicted distribution areas located on unsuitable habitat types. The occurrence data for generating the PDMs were obtained from The Atlas of Mexican Birds (Navarro, Peterson, & Gordillo‐Martinez, 2002). A total of 1,189 occurrence records for 12 species across the study area (Figure 1, inset b) were used to generate the PDMs; the number of records for each species with different location is shown in Table 1.

### 2.3 Prediction variables

The set of environmental prediction variables consisted of 19 bioclimatic variables obtained from the project WorldClim (Hijmans, Cameron, Parra, Jones, & Jarvis, 2005) and four topographic variables (Verdin & Greenlee, 1998) (Table 2). These bioclimatic variables, generated from monthly records of temperature and precipitation, are in raster format with a 0.01 × 0.01 degrees (aprox. 1 × 1 km) spatial resolution, and they represent annual trends, seasonality, and extremes values of climatic conditions (Hijmans et al., 2005). Topography variables included elevation above sea level (meters), slope (degrees), and aspect (degrees), and the topographic index, also known as a wetness index which is a function of the upstream contributing area and the terrain's slope (Moore, Grayson, & Ladson, 1991).

**Table 2:** Environmental prediction variables

Bioclimatic variables	
Annual mean temperature	bc1
Mean diurnal range (Mean of monthly (max temp–min temp))	bc2
Isothermality (P2/P7) (* 100)	bc3
Temperature seasonality (standard deviation *100)	bc4
Max temperature of warmest month	bc5
Min temperature of coldest month	bc6
Temperature annual range (P5–P6)	bc7
Mean temperature of wettest quarter	bc8
Mean temperature of driest quarter	bc9
Mean temperature of warmest quarter	bc10
Mean temperature of coldest quarter	bc11
Annual precipitation	bc12
Precipitation of wettest month	bc13
Precipitation of driest month	bc14
Precipitation seasonality (coefficient of variation)	bc15
Precipitation of wettest quarter	bc16
Precipitation of driest quarter	bc17
Precipitation of warmest quarter	bc18
Precipitation of coldest quarter	bc19
Topography	
Aspect	
Elevation	
Slope	
Topographic Index	

### 2.4 Species distribution modeling algorithms (SDMAs)

Three widely used and evaluated SDMAs were here applied and tested: (a) the Ecological Niche Factor Analysis (ENFA; Hirzel, Hausser, Chessel, & Perrin, 2002) is based on an adapted factor analysis that calculates habitat suitability functions from comparing the species environmental space against the global environmental space (Hirzel et al., 2002); (b) the Genetic Algorithm for Rule Set Production (Garp; Stockwell & Peters, 1999) applies a learning machine approach able to simultaneously generate and test a wide range of potential solutions, including range rules and logistic regression; and (c) Maximum Entropy (Maxent; Phillips, Anderson, & Schapire, 2006) which is used to estimate a target probability distribution by means of knowing the maximum entropy distribution; Maxent finds the most expanded distribution close to be uniform which is subjected to a set of restrictions that represent incomplete information about the target distribution (Phillips et al., 2006).

The application of Garp and Maxent included splitting each species' records in 75% for training and 25% for calculating model accuracy. Main modeling criteria and parameters for each SDMA are included inAppendix S1. The complete set of bioclimatic and topography variables were used for generating potential distribution models of species distributions, as determined by physical environment. One reason for not selecting a reduced set of variables was the nature of the ENFA approach which uses data redundancy for generating factors (Hirzel et al., 2001). Therefore, the three SDMAs were applied consistently with the same number of prediction variables. Another reason was the fact that most of species had enough number of records which would compensate the possibility of model overfitting (Guisan & Thuiller, 2005).

### 2.5 Species potential distribution models (PDMs) accuracy assessment

PDMs were evaluated by applying the Kappa Index (e.g., Allouche, Tsoar, & Kadmon, 2006; Congalton, 1991) and receiver operating characteristic (ROC; Guisan & Zimmermann, 2000; Fawcett, 2006). Besides Kappa, confusion matrices allowed calculation of accuracy and error measures such as global predictive success, sensitivity, specificity, and commission and omission errors (e.g., Forbes, 1995; Manel, Williams, & Ormerod, 2001; Robertson, Peters, Villet, & Ripley, 2003). The application of ROC complements potential weakness of the measures obtained from the error matrices (Fielding & Bell, 1997). Boundary vector data (shape files) about the species' geographic distribution (BirdLife International & NatureServe, 2015) were used as reference data to visualize the general distribution patterns shown by the generated SDMs.

### 2.6 Prediction thresholding

The test data, randomly separated by Maxent to perform internal accuracy assessment, were identified and independently used, along pseudoabsence data, for calculating both ROC and Kappa analyses for the three SDMAs. The lack of species absence data constrained this study to apply three presence‐only SDMAs. Therefore, a procedure was applied to generate the pseudoabsence data needed for calculating accuracy metrics: Areas predicted as species absence by the ENFA method were used to generate random points as pseudoabsences for testing models generated by Garp and Maxent. The pseudoabsences used to test ENFA models were generated in similar fashion but using absence areas predicted by Garp. Pseudoabsences for each species were generated by using the ArcView extension Random Point Generator (Jenness, 2005).

In order to obtain binary presence–absence models, threshold identification was achieved by two means: (1) locating the prediction value which maximizes the calculated Kappa for different prediction intervals and (2) locating on the ROC curve the prediction values in which sensitivity is maximized and 1‐Specificity is minimized (e.g., Liu, Berry, Dawson, & Pearson, 2005). Continuous (ENFA and Maxent) and discrete (Garp) predictions were assessed by calculating Kappa and ROC accuracy metrics for 5–7 different prediction value intervals, going from wide range of values (e.g., 20–100) to narrower (e.g., 80–100). A nonparametric two‐related samples test (Wilcoxon signed‐rank) was calculated to compare ROC‐ vs Kappa‐identified prediction thresholds.

### 2.7 Species occurrence data sampled on the field (FOD)

Species occurrence data sampled on the field (i.e., current species' presence; FOD) were used to test the predictive capabilities of each SDMA after PDMs were reduced to ADMs. A random stratified survey strategy was applied for collecting occurrence data across the region. Bird species were surveyed by the point count method (e.g., Hutto, Pletschet, & Hendricks, 1986): Within a 25 m radius, species are recorded during 15 min. Several count points (30 in average) were sampled at each of the 46 visited sites across the study area. Sampling started 15 min after sunrise, finishing 3–4 hr later. Distance among points was 200 m as minimum. Only areas dominated by the tropical dry forest were included for the survey. Field work was carried out during 3 years (2004, 2005, and 2006) in the months from June to August. These months are suitable for recording occurrences of bird species due that such period corresponds to the end of the dry season and part of the rainy season, the time when many species reproduce and they can be observed.

### 2.8 Species actual distribution models (ADMs)

Once prediction thresholds were determined so that presence/absence models (potential distribution) were obtained, and models were spatially reduced based on the documented information about each species' habitat preferences. The selected PDMs, among the three SDMAs for each species, corresponded to that for which the combination of thresholding and the accuracy values (kappa or AUC) generated the least overpredicted model.

The habitat type/species associations were identified by consulting databases online such as Neotropical birds—TheCornellLab of Ornithology (http://neotropical.birds.cornell.edu/portal/home); BirdLife International; IUCN‐RedList (http://www.iucnredlist.org/); and field guides and books on Neotropical birds (American Ornithologists, Union, 1998; Banks et al., 2005; Blake, 1977; Howell & Webb, 1995; Stotz et al., 1996). Then, an updated land use/land cover map (Velázquez et al., 2001) was used to reduce the PDMs to ADMs. Habitat types corresponded to a 1:250,000 scale vector map which was first rasterized to a 250‐m‐pixel‐size map and then overlaid to the 1‐km PDMs.

The ADMs' success for predicting species presence/absence was calculated by two means: Each species' presence point coverage (created from FOD) was rasterized to 1 × 1 km cell Grids, which were overlaid to the corresponding 1 × 1 km ASDM; then, the prediction success was calculated by the ratio [true presence/total sampled presence], where “true presence”= sampled presence matched by ASDM's predicted presence. A second procedure consisted in building 1‐km radius buffers around each sampled field site, with overlapping buffers being aggregated into single areas; predicted presence/absence values were extracted into the 1‐km buffers areas so that the pixel count was used to calculate prediction success: [true presence/total number of pixels included within sampled species presence buffers], where “true presence”= number of pixels where sampled presence was matched by ADM's predicted presence.

Finally, a species co‐occurrence model was built for purposes of identifying areas (i.e., ecoregions) with highest species richness estimates.

## 3 RESULTS

### 3.1 Key environmental prediction variables

Both ENFA and Maxent include tools for measuring the degree in which environmental variables contribute to generate the species' potential distribution models (PDMs). For ENFA, the scores associated with the factors suggest the level of importance that each environmental variable had in defining the model's marginality or tolerance, which are parameters that measure of how distinct and narrow are conditions where species occur, in relation to average conditions across the regions (Hirzel et al., 2002). Maxent on the other hand, runs Jackknife tests for identifying those variables which appears to have the most of useful information by themselves (Phillips et al., 2006).

**Figure 2:**
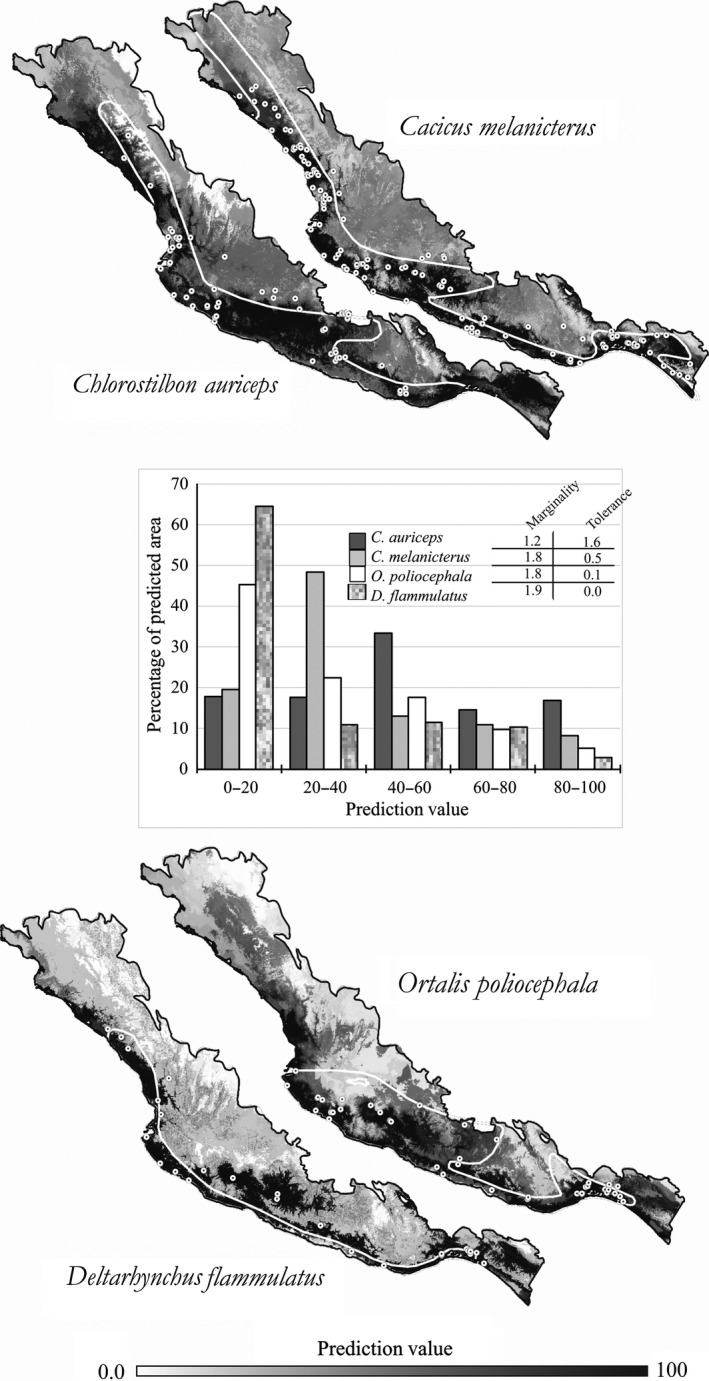
Four ENFA's potential species distribution models (PDMs), illustrating how the global Marginality and Tolerance indexes are reflected on model's characteristics. White dots are historic documented species occurrences (Navarro et al., 2002), used for model building. White line outlines represent the known species' geographic ranges, according to BirdLife International and NatureServe (2015). PDMs are displayed as a stretched continuous distribution with two standard deviations

All species differ from being distributed randomly across study region, according to the global marginality values for each species (M ≥ 1) (AppendixS2). The global tolerance values reflected a differential trend: Species such as *Deltarhynchus flammulatus*,* Granatellus venustus*,* Ortalis poliocephala,* and *Polioptila nigriceps* seem to show the most specialized distribution patterns (T∼0), while the rest of species showed intermediate specialization levels (T∼0.5) (AppendixS2). *Chlorostibon auriceps* seemed to be the least specialist species because of its highest tolerance value (1.6). To illustrate and compare the meaning of the Marginality and Tolerence indexes, Figure 2 shows ENFA's prediction models for four species with contrasting index values.

Variables that individually were important for explaining the distribution models' marginality included elevation, annual mean temperature (bc1), minimum temperature of coldest month (bc6), mean temperature of coldest quarter (bc11), precipitation seasonality (bc15), and precipitation of warmest quarter (bc18) (AppendixS2). These variables together had the highest marginality scores for all species modeled. For the tolerance factors, only three variables were important for explaining the species models' specialization (see AppendixS2): maximum temperature of warmest month (bc5), minimum temperature of coldest month (bc6), and temperature annual range (bc7).

Maxent's analysis showed also key variables predicting PDMs (Appendix S3). It is apparent that two variables, minimum temperature of coldest month (bc6) and mean temperature of coldest quarter (bc11), were individually the most important variables (using both training and test data) for more species (6–7 species) than the rest of variables. Other variables such as elevation, annual mean temperature (bc1), mean temperature of wettest quarter (bc8), and mean temperature of driest quarter (bc9) were important for fewer species.

### 3.2 PDM's accuracy assessment

Internal accuracy assessment, based on using a 25% partition of the museum occurrence data, consisted in running the ROC test for Maxent's PDMs and a Chi‐square test for Garp's PDMs. For the Maxent models, there were two species with 0.6 < AUC>0.8 (*Polioptila nigriceps* and *Vireo hypochryseus*), with the rest of species showing values of 0.8 < AUC>0.93. Based on a Chi‐square test that calculated the coincidence between test occurrence sites and model predictions, the best 10 Garp models, selected among 100 generated for each species (Anderson, Lew, & Peterson, 2003) showed uniformly *p*‐values < .01. Similarly, cross‐validation results obtained for ENFA models showed low F‐values for low model's prediction values and high F‐values for higher prediction values, which means that models were significantly different from random scenarios.

**Figure 3:**
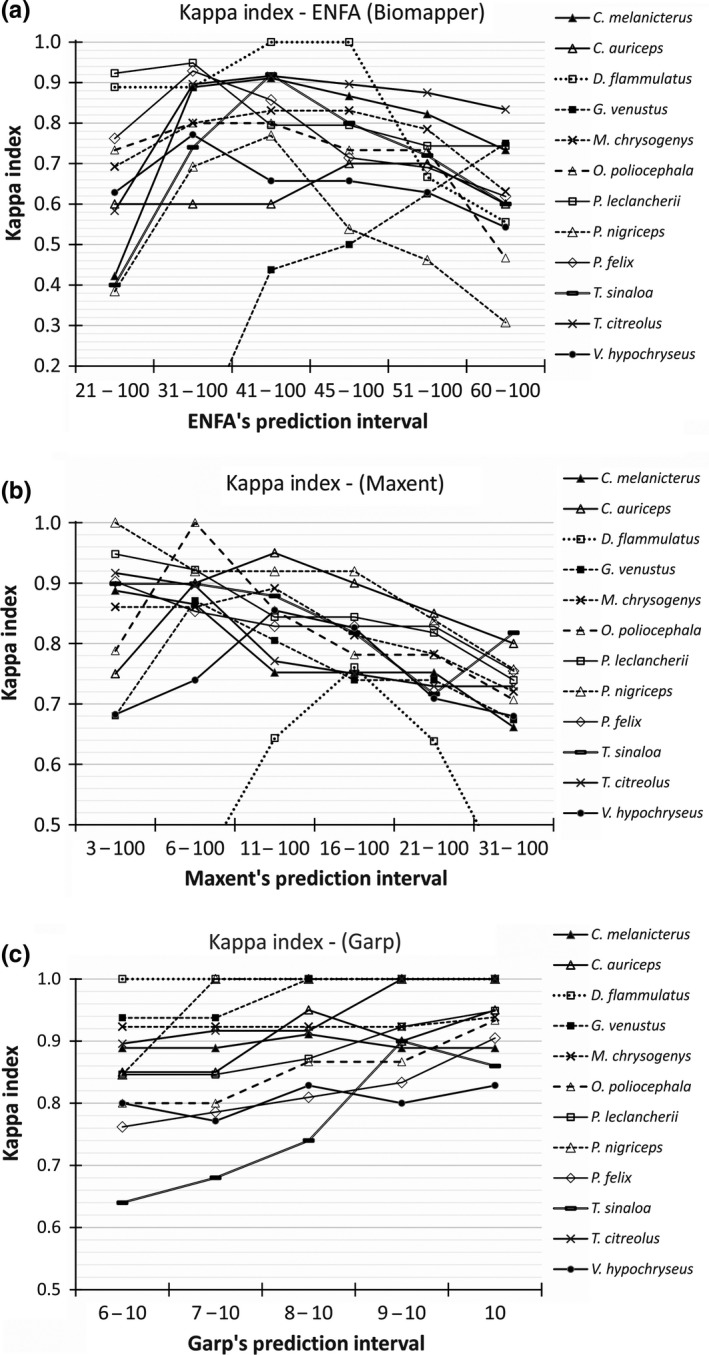
Kappa Index values, calculated for potential species distribution models of 12 endemic Mexican birds, generated by three species distribution modeling algorithms (SDMA) (ENFA, Garp, and Maxent). Kappa values were calculated for different prediction intervals (*x*‐axis) and plotted for each species

ROC and Kappa analyses, calculated outside the three SDM software, made possible to confirm the accuracy results just referred (i.e., high AUC values) and to identify variations in accuracy values as a function of intervals of prediction values (see Figure 3 for Kappa Index and Appendix S5 for ROC curves). For the ENFA and Maxent methods (Figure 3a and b, respectively), Kappa values across prediction intervals showed an apparent trend of low values for lower prediction intervals, then a rapid increase which could continue or be maintained for two or three intervals to finally drop in the higher prediction intervals. The Maxent method shows an exception to the pattern just described; for seven species, the Kappa value falls from the first prediction interval which is 3–100. Even though the prediction values for these two methods were scaled in the same range of values (0–100), it is apparent that prediction values behave differently between ENFA and Maxent. Kappa values for the Garp approach showed a very distinct pattern; these do not show a high variation as ENFA and Maxent, and they tend to have highest values in the last prediction interval with exception of only two species (Figure 3c).

### 3.3 Prediction thresholding

**Figure 4:**
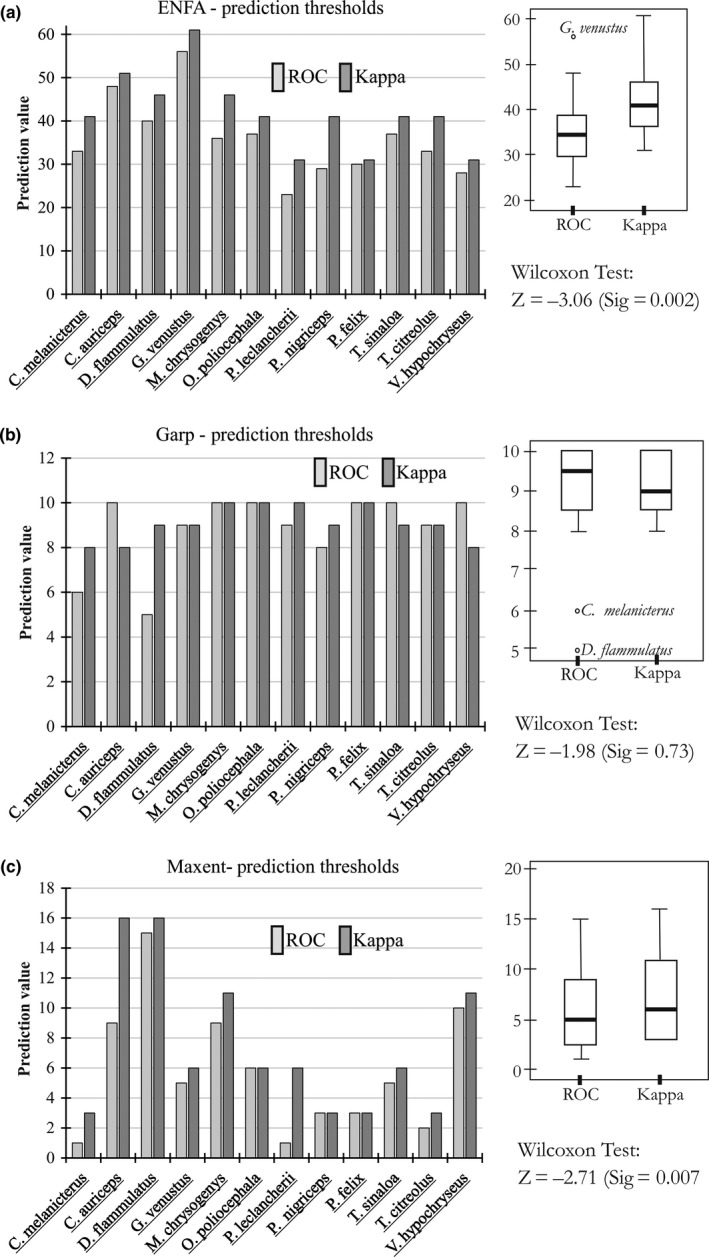
Identified prediction thresholds by species distribution modeling algorithms (SDMA), based on maximization of Kappa values and the nearest value to the Sensitivity= 1 and [1‐Specificity]= 0, on the ROC curve. Box plots show the variability and median prediction thresholds for each SDMA. Wilcoxon test (nonparametric test for two related samples) shows the results of comparing ROC thresholds vs Kappa thresholds for each SDM method. The null hypothesis is that there is no difference between the two groups

Based on the prediction threshold values, two criteria were applied to transform the PDMs from continuous (ENFA and Maxent) and discrete values (Garp) to presence/absence binary models: (1) maximization of Kappa Index and (2) maximization of Sensitivity (true positives rate) and minimization of [1‐Specificity] (false positives rate). ROC vs Kappa threshold values seem to be similar for each species' PDM (Figure 4), within the same SDMA (Figure 4). Species for which the two thresholds were more distant included: *Melanerpes chrysogenys* and *Polioptila nigriceps* (difference >10 units) for ENFA; *Deltarhynchus flammulatus* (difference= 4 units) for Garp; and *Chlorostilbon auriceps* and *Passerina leclancherii* (difference >5 units) for Maxent. Variability of threshold values among species within same SDMA was significant for both ROC and Kappa, but not for Garp, as revealed by the 2‐sample Wilcoxon test (see Figure 4).

The common geographic distribution pattern of species resembles the distribution of the main vegetation types of which this group of species is associated, the tropical dry forest (Appendix S6). It runs along the Pacific coast, from southern Sonora, going south through Pie de la Sierra, Sierras de las Costas de Colima y Jalisco, Costas del Sur, Cordillera Costera del Sur, reaching a depression in the state of Chiapas, with a significant inclusion into the continent at the Balsas depression (see Figure 1). A further comparison of PDMs' geographic patterns will be provided in the discussion.

### 3.4 Species' actual distribution models (ADMs)

**Figure 5:**
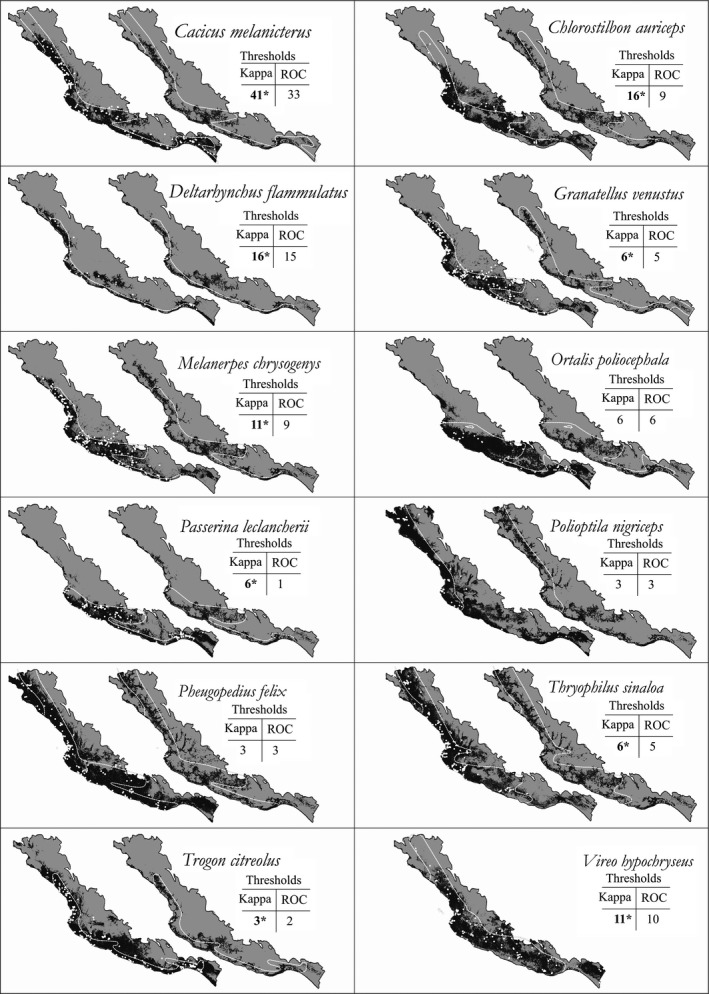
Binary (presence/absence) species actual distribution models (ADMs) and corresponding species potential distribution models (PDMs) models, for 12 endemic birds in Mexico. ADMs (left) and PDMs (right) are shown in pairs for each species, with black areas representing species predicted presence, and white lines representing known geographic distribution ranges (BirdLife International and NatureServe, 2015)

Actual distribution models (ADMs) (Figure 5), obtained for the 12 species of endemic birds, represent the spatially reduced versions of their corresponding potential distribution models (PDMs) (Figure 5), after eliminating those areas with unsuitable habitats as documented by habitat–species associations (Appendix S4). Even though the three species distribution modeling algorithms (SDMAs) showed similar high accuracy numbers (Figure 3), Maxent's PDMs were used to obtain the ADMs shown in Figure 5, on the basis that Maxent models consistently did not overpredict distribution patterns as much as Garp or even ENFA and showing lower conflicting distribution areas mapped. Notwithstanding the ADMs showing significantly constrained potential distribution areas (Figure 5), they follow the general geographic patterns shown by the PDMs but making distribution areas more fragmented.

The next step was to evaluate the level of spatial correspondence between these areas and the species occurrences sampled on the field (FOD), under both point and area approaches. When occurrence sites were rasterized to 1 × 1 km pixels, species with higher numbers of recorded presences included *Pheugopedius felix* and *Thryophilus sinaloa* with 92 different occurrences and *Melanerpes chrysogenys* with 87, while species with lowest numbers were *Polioptila nigriceps* (12) and *Chlorostilbon auriceps*,* Deltarhynchus flammulatus,* and *Granatellus venustus* with <30 (see Table 3). The distribution of the number of records per species in the FOD showed similar proportionality that the species museum datasets (*R* = .84; *p*‐value = .001), which is an indication that species occurrences were recorded on the field close to as expected by the historical species sampling across the region. However, the species *Cacicus melanicterus*,* Melanerpes chrysogenys*,* Trogon citreolus* have been still historically collected at higher proportions than this study's FOD, while the opposite happens with *Ortalis vetula*.

**Table 3:** Cross‐tabulation between species occurrences recorded on the field and the areas predicted as actual presence/absence

	Site evaluation				Area evaluation			
Species	Total (1x1 km) presences	True presence	False absence	Success Rate%	Total (buffer) presence cells	True presence cells	False absence cells	Success Rate%
*Cacicus melanicterus*	62	35	27	49.4	150	86	64	57.3
*Chlorostilbon auriceps*	23	15	8	73.5	63	47	16	74.6
*Deltarhynchus flammulatus*	26	22	4	60.7	67	56	11	83.6
*Granatellus venustus*	29	20	9	81.8	75	57	18	76.0
*Melanerpes chrysogenys*	87	58	29	64.3	197	134	63	68.0
*Ortalis poliocephala*	58	39	19	64.3	156	102	54	65.4
*Passerina leclancherii*	78	50	28	61.2	184	121	63	65.8
*Polioptila nigriceps*	12	6	6	55.5	28	15	13	53.6
*Pheugopedius felix*	92	61	31	64.4	197	139	58	70.6
*Thryophilus sinaloa*	92	60	32	68.9	187	126	61	67.4
*Trogon citreolus*	70	49	21	64.4	170	112	58	65.9

Where: Total (1x1 km) presences= Total number of 1x1 km cells, recorded as species presence on the field; True presence= model's predicted presence and recorded presence on the field; False absence= model's predicted absence and recorded presence on the field; Total (buffer) presence cells= pixels within 1‐km radius buffer areas around sites where species were found present on the field; True presence cells= pixels within 1‐km radius buffer areas around sites where species were found present on the field and model's predicted presence; False absence cells= pixels within 1‐km radius buffer areas around sites where species were found present on the field and model predicted absence.

ADMs were successful when predicting species' presence in an 66% average (std. deviation = 8.1) of the field occurrence sites (1 × 1 km cell) for all species, with the highest for *Deltarhynchus flammulatus* (84%) and the lowest for *Polioptila nigriceps* (50%) (Table 3). By generalizing the calculation of the spatial correspondence between ADMs' presence/absence values and the FOD's 1‐km radius buffer areas, the prediction success among species were similar to the 1x1 km site evaluation (see Table 3); the average success of the former was 68% (std. deviation= 8.0). The main reason for applying an area evaluation method was to validate the ADMs based on their contiguous configuration of both the ADMs' cell values (presence/absence) and the buffered area resulting from the proximity among FOD's sites.

Tropical dry forest ecoregions (Balsas, Jalisco, Sinaloan, and Southern Pacific) included 60% of low (1–4) and 90% of the medium (5–8 spp.) and high (9–11 spp.) species co‐occurring areas (see Figure7). A number of temperate (e.g., pine and pine–oak) ecoregions accounted for just an average of 7.5% (Std. dev. = 2.8) of the species co‐occurrence's total area. Among the four tropical dry forest ecoregions, the Balsas' tropical dry forest included the highest proportion of highest species co‐occurrence (9–12 spp.), followed by the Southern Pacific's tropical dry forest ecoregion. However, high species co‐occurrence uniformly distributes across the Pacific slope as a rather narrow strip. The Balsas' tropical dry forest ecoregion appears as the wider region where there is highest species co‐occurrence (see Figure7).

## 4 DISCUSSION

Distribution models for the group of 12 endemic bird species made evident that species are not constrained to be distributed within the tropical dry forest, which is the vegetation type documented as these species' main habitat (Vega Rivera et al., 2010). The physiographic provinces, used to define the whole study area, adequately included the different habitat types where this group of species is distributed. Besides temperate forests, other habitat types where species can be distributed include the tropical moist montane/lowland and even human‐transformed environments (e.g., The IUCN Red List of Threatened Species, 2016). For instance, altitudinal limits for some species can reach the 2,600 m (e.g., *Chlorostilbon auriceps*), which would suggest their presence in other habitat types (e.g., temperate) besides the tropical habitats.

### 4.1 Key prediction variables

Identification of prediction variables as important in the generation of species' potential distribution models (PDMs) contributes to describe the characteristics of each SDM.

ENFA's global Marginality (M) and Tolerance (T) indexes provide measures of how different and specialized, respectively, the modeled suitable habitats are in relation to the whole region. The meaning of such indexes would be reflected on how prediction values are distributed across the model's range of values (see histogram in Figure 2). For instance, PDMs for both species *D. flammulatus* and *O. poliocephala* showed a specialized distribution across the region (T∼ 0), which can be visualized by looking at the more accentuated differentiation among the range of prediction values, as compared to *C. auriceps* with the highest Tolerance, and even when compared to *C. melanicterus* with a medium Tolerance value. Both the histogram and maps in Figure 2 show such differences; for instance, the *C. auriceps* model tend to have a more uniform prediction values distribution which is reflected on a less contrasting range of gray shades in the map.

Even though there was high variability of variables identified as important among the 12 species models, ENFA and Maxent coincided identifying minimum temperature of coldest month (bc6) and mean temperature of coldest quarter (bc11) as two of the most important prediction variables for the largest number of species, followed by annual mean temperature (bc1) and elevation. Unfortunately, this information is not conclusive because of the difficulties in finding and applying a threshold from which differentiate the most relevant variables identified as important in building the SDMs. There is also the fact that variables may show high levels of correlation among themselves.

### 4.2 Species potential distribution models (PDMs)

Species' potential distribution models (PDMs) were generated under the assumption that species geographic ranges can initially be identified by significant associations between a relatively unchanged physical environment (i.e., bioclimatic and topography variables) and the historical species' occurrences. The species occurrence dataset used (Navarro et al., 2002) is a part of one of the most comprehensive and scientifically recognized datasets documenting the distribution of Mexican birds (Peterson et al., 2015). The PDMs generated by the three SDMAs reflect the different levels in which habitats are suitable for each of the 12 species (Hirzel & Le Lay, 2008), based on the physical conditions associated with those places where species have been documented to occur (see Appendix S6).

Generated PDMs tend to resemble the 12 species' geographic distribution patterns documented by the BirdLife International & NatureServe (2015): Species distribute along the western Pacific slope, on a strip that varies in width depending how far species get into mountainous environments (Sierra Madre Occidental; Sierras de Jalisco y Colima, Cordillera Costera del Sur, and Sierras del Sur de Chiapas) (see Figure 1). Because of the strong association of the 12 species with environments where the tropical dry forest distributes, most of PDMs included deeper inland areas corresponding to the Balsas and Tepalcatepec physiographic depressions, where the tropical dry forest is the dominant vegetation formation.

A visual revision of the PDMs (Appendix S6) allows the identification of several general characteristics and differences among models. It is apparent that Garp tend to overestimate distributional areas (Elith & Graham, 2009), although Garp models consisted of a much narrower range of values (1–10 vs 0–100 of ENFA and Maxent) so that highest prediction values (e. g., 8–10) tend to visually occupy most of predicted areas. Distribution latitudinal limits, as suggested by the location of species occurrences sites (SOS) used in this study and the documented species' range maps (DRM) (BirdLife International & NatureServe, 2015), seem to be modeled with some discrepancies among the three species distribution modeling algorithms (SDMAs). For instance, for four species, *Chlorostilbon auriceps*,* Melanerpes chrysogenys*,* Pheugopedius felix,* and *Thryophilus sinaloa*, both DRM and SOS suggest these species' southern distributions would not be located beyond the Tehuantepec Isthmus (Figure 5); however, only Maxent seems to better model such a pattern. Similarly, even though the three SDMAs tend to constrain the northern distribution for *Ortalis policephala* and *Passerina leclancherii* to the southern Bahia de Banderas (as suggested by the DRM and SOS information), the Maxent's models also better reproduce such condition (Figure 5). Finally, for *Polioptila nigriceps* DRM and SOS seem to suggest contradictory information about the species' southern distribution limits; while the former puts it on the state of Colima, the later includes sites located on the Tehuantepec Isthmus. Again, what looks like an overfitted Maxent model seems to better constrain the species distributional areas.

These observations make evident the need for investigating objective and robust methods to identify prediction thresholds that would provide models with the most possible certainty.

### 4.3 PDMs accuracy and prediction thresholds

It is important to point out again that this study's main objective is not to evaluate which of the three applied SDMAs is the most accurate. The main concern is to test the application of SDMAs for generating species actual distribution models (ADMs), based on applying objective criteria for selecting prediction thresholds and using documented habitat–species associations for reducing the PDMs to approximate versions of ADMs.

As expected, the three SDMAs generated accurate models, as revealed by the accuracy measures calculated from an independent set of documented occurrence data; only for two species, Maxent's models obtained AUC< 0.80. However, in order to transform continuous (ENFA and Maxent) and ordinal (Garp) models to binary (presence/absence) models, ROC curves and Kappa tests needed to be performed outside each of the SDM software. These tests were applied to assess the performance of partial prediction values of each SDMA and not necessarily for comparing among themselves.

Appendix S5 shows the PDMs' resulting ROC curves; all models showed ROC curves with shapes that suggest perfect discrimination among prediction values, with very high AUC values. Higher prediction values correspond to points on the curve where the false positive rate (FPR, *x*‐axis) approaches to cero and the true positive rate (TPR, *y*‐axis) approaches to one. Points located on the ROC curve further away from the corner (0, 1) correspond to lowest prediction values. The point on each ROC curve, located closest the upper left corner, was identified as a prediction threshold because it suggests the highest accuracy (Zweig & Campbell, 1993).

Kappa values for partial prediction values of PDMs generated by ENFA and Maxent showed clear patterns in which there is an increase from low prediction values to reach a maximum and then decrease at highest prediction value intervals (Figure 3). ENFA reproduces this pattern better than Maxent because this later seemed to have generated more overfitted models which caused that for some species Kappa values decreased from the lowest prediction interval. Garp models showed distinct Kappa patterns; these were more difficult to differentiate because of the narrower range of prediction values (just 5 intervals), but the highest Kappa values corresponded to highest prediction values (9–10).

Identified Kappa and ROC thresholds for each species within SDMA were consistently equivalent, which suggest the use of both accuracy parameters seems a robust criterion for deciding the minimum value from which continuous models can be transformed to binary (presence/absence) distribution models. Presence/absence models enable to reduce prediction uncertainty, making possible to use them for practical analysis such as richness calculations or even climate change predictions (e.g., Araújo, Thuiller, & Pearson, 2006; Luoto, Heikkinen, Poyry, & Saarinen, 2006). However, when the median threshold was calculated for the 12 species, the comparison Kappa vs ROC was significantly different for Maxent and ENFA but not for the Garp models (Figure 4). Given these differences that seem slight at first glance but statistically significant, the selection between Kappa‐ and ROC‐derived thresholds should be further assessed when groups of species are analyzed.

Examination of the binary models obtained from applying both ROC and Kappa thresholds made evident that after thresholding, some of the resulting outcomes showed traits which may help to decide which SDM's threshold to use. For some species, models resulted in very similar presence/absence PDMs, such as *Cacicus melanicterus*,* Granatellus venustus*,* Melanerpes chrysogenys*,* Passerina leclancherii,* and *Trogon citreolus*. However, for other species thresholding revealed apparent overpredictions: *Ortalis poliocephala* (Maxent and ENFA), *Polioptila nigriceps* (Maxent), *Pheugopedius felix,* and *Thryophilus sinaloa* (ENFA and Maxent). It became clear the apparent overprediction of Garp was not as evident and exclusive as this approach.

Previous work, like Liu et al. (2005) and Jiménez‐Valverde and Lobo (2007), have found that thresholding using the ROC curve (sensitivity/specificity criteria) produced good results for thresholding continuous prediction models. However, different from this study, they found Kappa did not work as well. In this study, Kappa's maximization worked as well as the ROC curve and even better because the Kappa's thresholds were uniformly higher than ROC's; therefore, attenuating the overprediction identified by the expert opinion criteria. The selection of the appropriate threshold would depend on the research questions and design (Loiselle et al., 2003). Careful and specific thresholding of continuous SDMs is required for avoiding that arbitrary thresholds may obscure important biological traits and even mislead about patterns of niche overlap (Warren, Glor, Turelli, & Funk, 2008).

### 4.4 Species' actual distribution models (ADMs)

It is rather apparent the contrast in the extent and size of predicted areas when PDMs are compared with ADMs (Figure 5). PDMs are drastically reduced as a result of eliminating areas with land use/land cover unsuitable for each species. In this modeling exercise, the selection of habitat types suitable for individual species was implemented based on considering no‐human‐transformed landscapes only. Within our group of 12 bird species, there are some which in fact are known for using at some extent transformed environments. However, because the significance of those transformed environments is unknown, this study's main concern was the actual availability of original habitats on which these endemic species depend.

**Figure 6:**
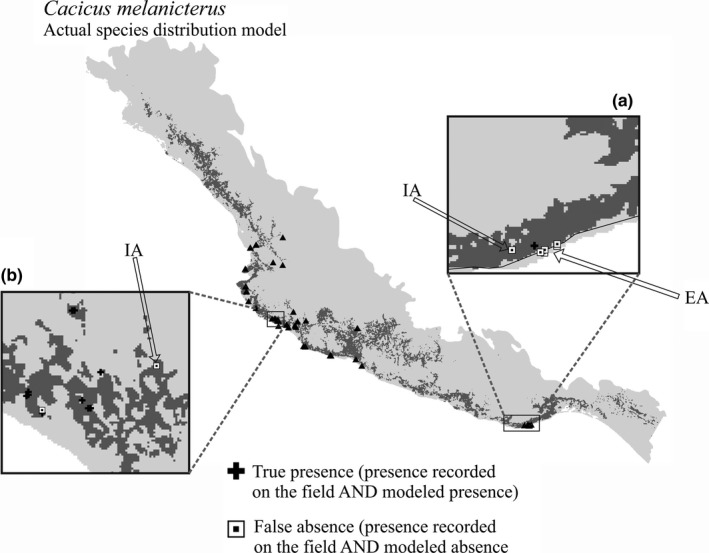
Influence of spatial resolution and spatial context on the correspondence between species actual distribution models (ADMs) and species occurrence sampled on the field. True presences (black cross) represent species presence recorded on the field and modeled as species presence. False absences (white squares) represent species presence recorded on the field and modeled absence. IA are examples of species presences recorded on the field but predicted as absence, in a spatial context dominated by predicted presence. EA are examples of species presences recorded on the field but predicted as absence, where there exist an edge effect related to issues of data spatial resolution

After assessing the spatial correspondence between the ADMs' predicted presence and the species occurrence data sampled on the field (FOD), it was apparent that differences in the spatial resolution between these two datasets suggested an explanation for some of the mismatches obtained. For instance, the 1‐km raster bioclimatic and topographic data fail to capture areas sampled along the coast line (see Figure 6). Another interesting situation occurred when a FOD (i.e., species occurrence) corresponded to a model's predicted species absence, but at the same time such a location also corresponded to areas highly dominated by the model's predicted presence (see Figure 6).

Because of these conditions concerning to the spatial resolution and spatial context, a conservative “area‐based” approach (1‐km radius buffers) was undertaken to test potential variations in the spatial correspondence between ADMs and FOD. The application of the “area‐based” approach basically meant the addition of two or three more pixels next to each pixel corresponding to the 1 × 1 km FOD' sites. The “area‐based” approach incorporated 2–4 times more (contextual) pixels than the “site” approach (see Table 3, columns “Total (1x1 km) presences” and “Total (buffer) presence cells”); both correspondence analyses were carried out on a pixel to pixel basis. The slight increase in prediction success when “area‐based” approach is applied seemed to capture, at some degree, the issues derived from managing data with different spatial resolutions combined with the pixel configuration associated with the SDMs, as described above.

Another important factor determining the SDMs' prediction success would be the potential changes in the land use/land cover configuration; the digital land use/land cover map, used to reduce PDMs to ASDMs, was elaborated using 1999–2000 data and the field work collecting species occurrence data was carried out 4–5 years later. The location of FOD's points for some species, on small areas predicted as absence but within a matrix of predicted presence areas, supports the hypothesis of changing landscapes (Figure 6).

**Figure 7:**
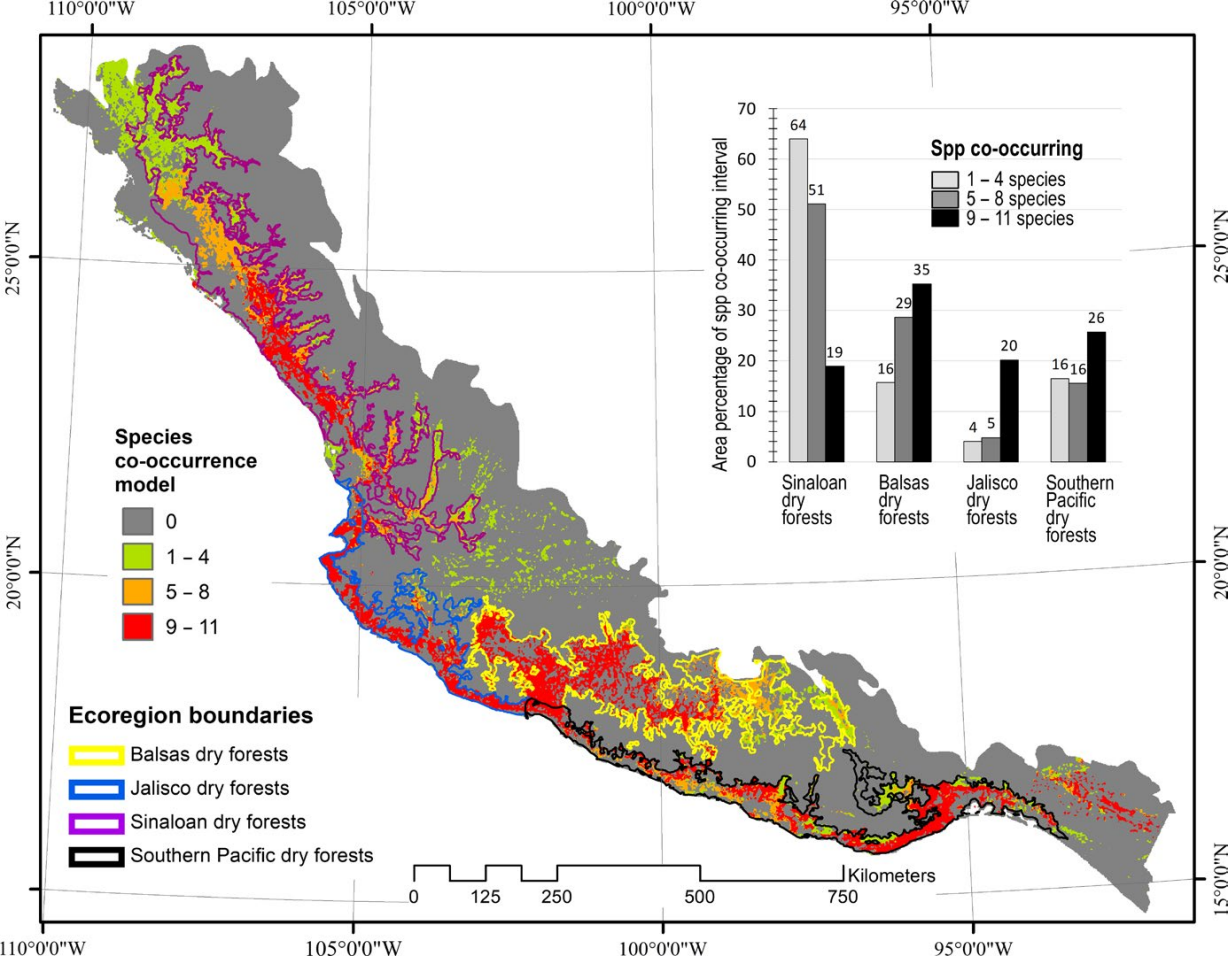
Species co‐occurrence model obtained by adding actual binary distribution models for eleven species of endemic bird species. Three co‐occurrence intervals are shown in green, orange, and red, while ecoregions are delineated by four color lines. The bar graph shows the percentage of each co‐occurrence interval located within four main ecoregions

The provided species co‐occurrence model (Figure 7) may be used for prioritizing areas with high co‐occurrence of endemic species of Mexican birds. Even though highest species co‐occurrence areas distribute along Mexico's western slope as relatively narrow strips, significant portions of these seem continuous (middle Sinaloan tropical dry forest, Jalisco tropical dry forest coast, and southeastern Southern Pacific tropical dry forest). There is only a small biosphere reserve (~130 km^2^) for protecting these areas. On the interior land (western half of the Balsas tropical dry forest ecoregion), highest species co‐occurrence areas are more extensive; here, there exists the Zicuirán Infiernillo Biosphere Reserve, which is a much larger area (~2,650 km^2^). Further studies are necessary for assessing the potential of small groups of species like this study's, as indicators of biodiversity, given the absence of a natural protected areas network for protecting the tropical dry forest's biodiversity in Mexico.

## CONFLICT OF INTEREST

None declared.
