# Supplementary material for: Validating distribution models for twelve endemic bird species of tropical dry forest in western Mexico
Source: Ecol Evol. 2017 Aug 19;7(19):7672–86. doi: 10.1002/ece3.3160 (PMC5632607; doi:10.1002/ece3.3160)
Supplement: Supplementary file 1 [file ECE3-7-7672-s001.docx]

| Appendix S1. Species distribution modeling algorithms (SDMA) main modeling criteria and parameters. Accuracy assessment used 25% of records, therefore 75% were used for training. | | |
| --- | --- | --- |
| SDMA | Prediction/Optimization parameters | Other parameters |
| ENFA | Median as Habitat Suitability Algorithm | Number of factors chosen by broken stick; scaling by stretching to 100; 10 partitions for cross-validation |
| GARP | Convergence limit: 0.001  Max. iterations: 100  Rule types: Atomic, range, negated and logistic | Best subset selection based on 5% omission, 20 models under hard omission threshold, 100 models as maximum and 50% commission threshold; |
| Maxent | Feature types used: Linear, Quadratic, Product, Threshold, Hinge; 1.0 Regularization multiplier; 500 max. iterations | Receiving Operating Characteristic (ROC) curves. |
