# Supplementary material for: Validating distribution models for twelve endemic bird species of tropical dry forest in western Mexico
Source: Ecol Evol. 2017 Aug 19;7(19):7672–86. doi: 10.1002/ece3.3160 (PMC5632607; doi:10.1002/ece3.3160)
Supplement: Supplementary file 2 [file ECE3-7-7672-s002.docx]

| Appendix S2. Important environmental variables predicting the species distribution models for 12 endemic bird species in western Mexico, generated by the ENFA approach. M= marginality factor; T= Tolerance factor. | | | | | | | | | | | | |
| --- | --- | --- | --- | --- | --- | --- | --- | --- | --- | --- | --- | --- |
|  | *Casicus melanicterus* | *Chlorostibon auriceps* | *Deltarhyncus flammulatus* | *Granatelus venustus* | *Mela0nerpes chrysogenys* | *Ortaln sis poliocephala* | *Passerina leclancheri* | *Polioptila nigriceps* | *Pheugopedius felix* | *Thryophilus Sinaloa* | *Trogon citreolus* | *Vireo hypochryseus* |
| Aspect |  |  |  |  |  |  |  |  |  |  |  |  |
| Elevation | **C** |  | **C** | c-t |  |  |  | **C**-**T** | c-t | c | c-**T** |  |
| Slope |  |  |  |  |  |  |  |  |  |  |  |  |
| Topographic Index |  |  |  |  |  |  |  |  |  |  |  |  |
| bc1 | c |  | **C** | c | c-t | c | c-t | t |  | **C** |  |  |
| bc2 | t |  | t | t |  |  |  |  |  |  |  |  |
| bc3 |  | **C**-t | t |  |  |  |  |  |  |  |  | t |
| bc4 |  | c |  |  |  | c |  |  |  |  |  | c |
| bc5 |  |  |  |  |  |  |  |  |  |  |  |  |
| bc6 | **C** | c-t | **C**-t | **C-T** | **C-T** | **C-T** | c-t |  | **C**-t |  | **C**-t | c |
| bc7 |  | c | **T** |  |  | t |  |  | t |  |  | **C** |
| bc8 | **C-T** |  | c | c | c-t |  |  | c-t |  | **C**-t | c-**T** |  |
| bc9 | c-**T** |  | c | T | c-t |  | c-t |  |  | c-**T** |  |  |
| bc10 |  |  |  |  |  |  |  | c-t |  | **C**-t |  |  |
| bc11 | c-t | **C-T** | c | **C**-**T** | **C-T** | **C**-t | **C-T** | t | c | c-**T** |  |  |
| bc12 |  | c-t |  |  |  |  |  |  |  |  |  | t |
| bc13 |  |  |  |  |  |  |  |  |  |  |  |  |
| bc14 |  |  |  |  |  |  |  | c |  |  |  |  |
| bc15 |  |  |  |  | t |  |  |  | c | **C-T** |  |  |
| bc16 |  | c |  |  |  |  |  |  |  |  |  | **T** |
| bc17 |  |  |  |  |  |  |  |  |  |  |  |  |
| bc18 |  |  |  |  |  |  |  |  | **T** | c-**T** |  |  |
| bc19 |  |  |  |  |  |  |  |  |  |  |  |  |
