# Supplementary material for: Validating distribution models for twelve endemic bird species of tropical dry forest in western Mexico
Source: Ecol Evol. 2017 Aug 19;7(19):7672–86. doi: 10.1002/ece3.3160 (PMC5632607; doi:10.1002/ece3.3160)
Supplement: Supplementary file 3 [file ECE3-7-7672-s003.docx]

| Appendix S3. Important environmental variables predicting the species distribution models for 12 endemic bird species in western Mexico, generated by the Maxent approach. | | | | | | | | | | | | |
| --- | --- | --- | --- | --- | --- | --- | --- | --- | --- | --- | --- | --- |
|  | *Casicus melanicterus* | *Chlorostibon auriceps* | *Deltarhyncus flammulatus* | *Granatelus venustus* | *Melanerpes chrysogenys* | *Ortalis poliocephala* | *Passerina leclancheri* | *Polioptila nigriceps* | *Pheugopedius felix* | *Thryophilus sinaloa* | *Trogon citreolus* | *Vireo hypochryseus* |
| Number of factors | 12 | 5 | 4 | 8 | 10 | 5 | 8 | 3 | 10 | 10 | 13 | 14 |
| Global Marginality | 1.8 | 1.2 | 1.9 | 1.5 | 1.5 | 1.8 | 1.9 | 1.0 | 1.1 | 1.1 | 1.8 | 0.8 |
| Global Tolerance | 0.5 | 1.6 | 0.0 | 0.3 | 0.4 | 0.1 | 0.3 | 0.05 | 0.5 | 0.4 | 0.5 | 0.5 |
| Aspect |  |  |  |  |  |  |  |  |  |  |  |  |
| Elevation | M |  |  | M |  |  |  | M |  | M | M |  |
| Slope |  |  |  |  |  |  |  |  |  |  |  |  |
| Topographic Index |  |  |  |  |  |  |  |  |  |  |  |  |
| bc1 |  |  | M | M | M |  | M |  |  |  | M |  |
| bc2 |  |  |  |  |  | M |  |  |  |  | M |  |
| bc3 |  |  |  |  |  |  |  |  |  |  |  |  |
| bc4 |  |  |  |  |  |  |  |  |  |  |  | M |
| bc5 | T | T | T | T | T | T | T | T | T | T | T | T |
| bc6 | M-T | T | M-T | M-T | M-T | M-T | M-T | T | T | T | M-T | M-T |
| bc7 | T | T | T | T | T | MT | T | T | T | T | T | M-T |
| bc8 |  |  |  |  |  |  |  | M |  |  |  |  |
| bc9 |  |  |  | M |  |  | M |  |  |  |  |  |
| bc10 |  |  |  |  |  |  |  | M |  |  |  |  |
| bc11 |  |  | M | M | M | M | M |  |  |  | M |  |
| bc12 |  |  |  |  |  |  |  |  |  |  |  |  |
| bc13 |  |  |  |  |  |  |  |  |  |  |  |  |
| bc14 |  |  |  |  |  |  |  |  |  |  |  |  |
| bc15 |  | M |  |  |  |  |  | M | M | M |  |  |
| bc16 |  |  |  |  |  |  |  |  |  |  |  |  |
| bc17 |  |  |  |  |  |  |  |  |  |  |  |  |
| bc18 |  | M |  |  |  |  |  | M | M | M |  |  |
| bc19 |  |  |  |  |  |  |  |  |  |  |  |  |
