# Supplementary material for: Validating distribution models for twelve endemic bird species of tropical dry forest in western Mexico
Source: Ecol Evol. 2017 Aug 19;7(19):7672–86. doi: 10.1002/ece3.3160 (PMC5632607; doi:10.1002/ece3.3160)
Supplement: Supplementary file 4 [file ECE3-7-7672-s004.docx]

| Appendix S4. Associations between habitat types (Inventario Forestal 2000) and 12 endemic bird species, obtained from scientific literature (see Methods section) and expert opinion. | | | | | | | | | | | | |
| --- | --- | --- | --- | --- | --- | --- | --- | --- | --- | --- | --- | --- |
|  | *C. melanicterus* | *C. auriceps* | *D. flammulatus* | *G. venustus* | *M. chrysogenys* | *O. poliocephala* | *P. leclancherii* | *P. nigriceps* | *P. felix* | *T. sinaloa* | *T. citreolus* | *V. hypochryseus* |
| Agriculture |  |  |  |  |  |  |  |  |  |  |  |  |
| Cultivated grassland |  |  |  |  |  |  |  |  |  |  |  |  |
| Seasonal agriculture |  |  |  |  |  |  |  |  |  |  |  |  |
| Juniper forest |  |  |  |  |  |  |  |  |  |  |  |  |
| Abis forest |  |  |  |  |  |  |  |  |  |  |  |  |
| Pine forest |  |  |  |  |  |  |  |  |  |  |  |  |
| Open short forest |  |  |  |  | **X** |  |  |  |  |  |  |  |
| Pine-oak forest |  |  |  |  |  |  |  |  |  |  |  |  |
| Oak forest |  |  |  |  |  |  |  |  |  |  |  |  |
| Cloud forest |  |  |  |  |  |  |  |  |  |  |  |  |
| Tropical evergreen forest |  |  |  |  |  | **X** |  |  |  |  |  |  |
| Tropical semi-evergreen forest |  |  |  |  |  | **X** |  |  |  |  |  |  |
| Tropical deciduous and semi-deciduous forest | **X** | **X** | **X** | **X** | **X** | **X** | **X** | **X** | **X** | **X** | **X** | **X** |
| Tropical short deciduous and semi-deciduous forest | **X** | **X** | **X** | **X** | **X** | **X** | **X** | **X** | **X** | **X** | **X** | **X** |
| Semi-tropical shrub | **X** | **X** | **X** | **X** | **X** | **X** | **X** | **X** | **X** | **X** | **X** | **X** |
| Thorn forest | **X** | **X** | **X** | **X** | **X** | **X** | **X** | **X** | **X** | **X** | **X** | **X** |
| Mezquite |  | **X** |  | **X** |  |  | **X** | **X** |  |  |  | **X** |
| Semi-desert scrub |  | **X** |  | **X** |  |  | **X** | **X** |  |  |  | **X** |
| Desert sand vegetation |  | **X** |  |  |  |  | **X** | **X** |  |  |  | **X** |
| Chaparral |  | **X** |  | **X** |  |  | **X** | **X** |  |  |  | **X** |
| Natural grassland |  |  |  |  |  |  |  |  |  |  |  |  |
| Savanna |  |  |  |  | **X** |  |  |  |  |  |  |  |
| Introduced grassland |  |  |  |  |  |  |  |  |  |  |  |  |
| Mangrove |  |  |  |  |  |  |  |  |  | **X** |  |  |
| Wetlands (other) |  |  |  |  |  |  |  |  |  |  |  |  |
| Gallery forest |  |  |  |  | **X** |  |  | **X** |  | **X** | **X** | **X** |
| Palm forest |  |  |  |  |  |  |  |  |  |  |  |  |
| Halophytic and gypsophyllous vegetation |  |  |  |  |  |  |  |  |  |  |  |  |
| Costal sand dune vegetation |  |  |  |  |  |  |  |  |  |  |  |  |
| Bare soil |  |  |  |  |  |  |  |  |  |  |  |  |
| Human settlement |  |  |  |  |  |  |  |  |  |  |  |  |
| Water bodies |  |  |  |  |  |  |  |  |  |  |  |  |
