# Supplementary material for: Validating distribution models for twelve endemic bird species of tropical dry forest in western Mexico
Source: Ecol Evol. 2017 Aug 19;7(19):7672–86. doi: 10.1002/ece3.3160 (PMC5632607; doi:10.1002/ece3.3160)
Supplement: Supplementary file 5 [file ECE3-7-7672-s005.docx]

| Appendix S5. ROC-Curves for the potential species distribution models of 12 endemic Mexican birds, generated by three species distribution modeling algorithms (SDMA) (ENFA, Garp and Maxent). Area under the curve (AUC) values are provided as a measure of the accuracy. For each species a set of independent occurrence data and pseudo-absence data were used to calculate the ROC-Curves. The axis-y represents the true positive rate (TPR) or sensitivity and the axis-x is false positive rate (FPR) or (1-specificity). |
| --- |
| 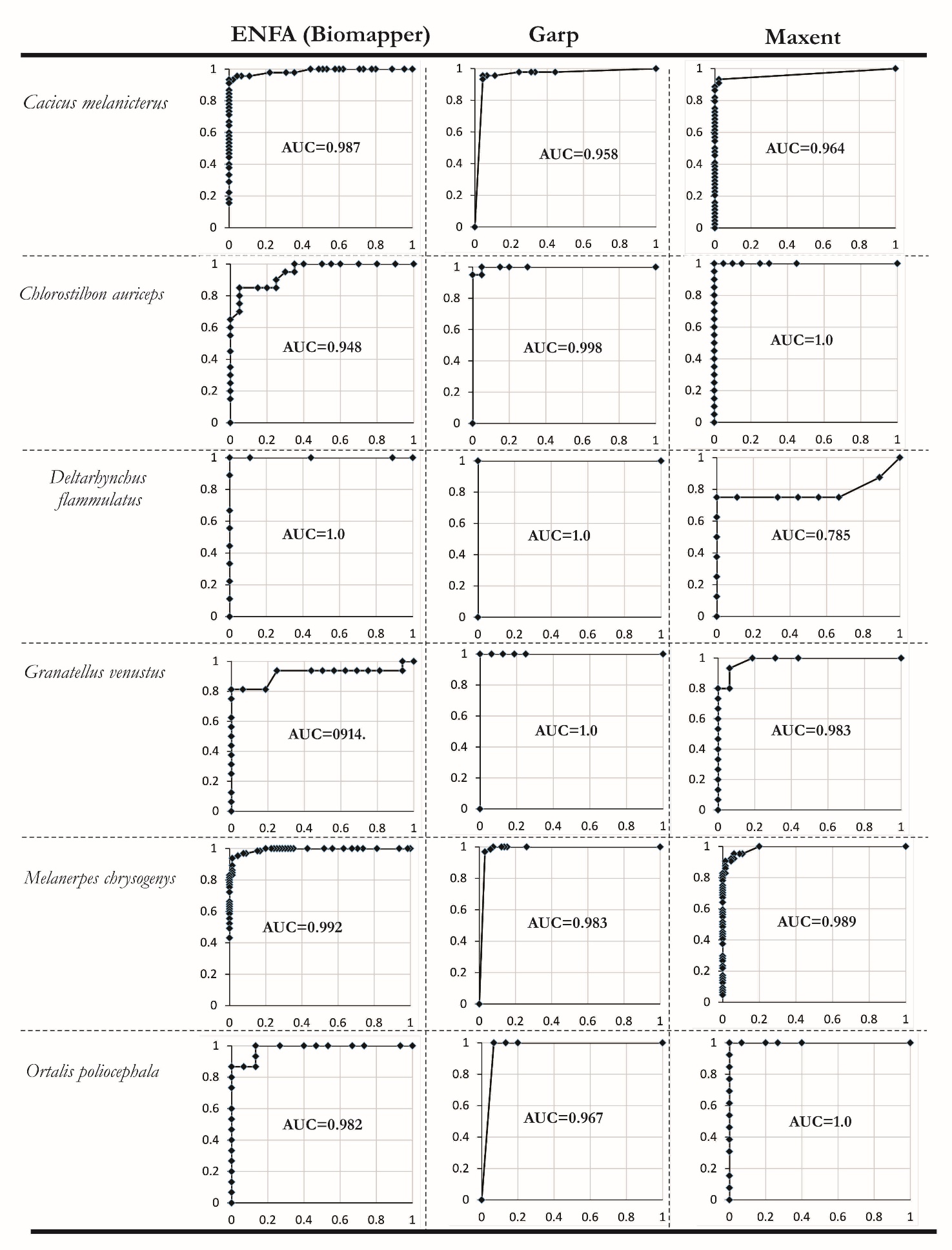 |

| Appendix S5 Cont. |
| --- |
| 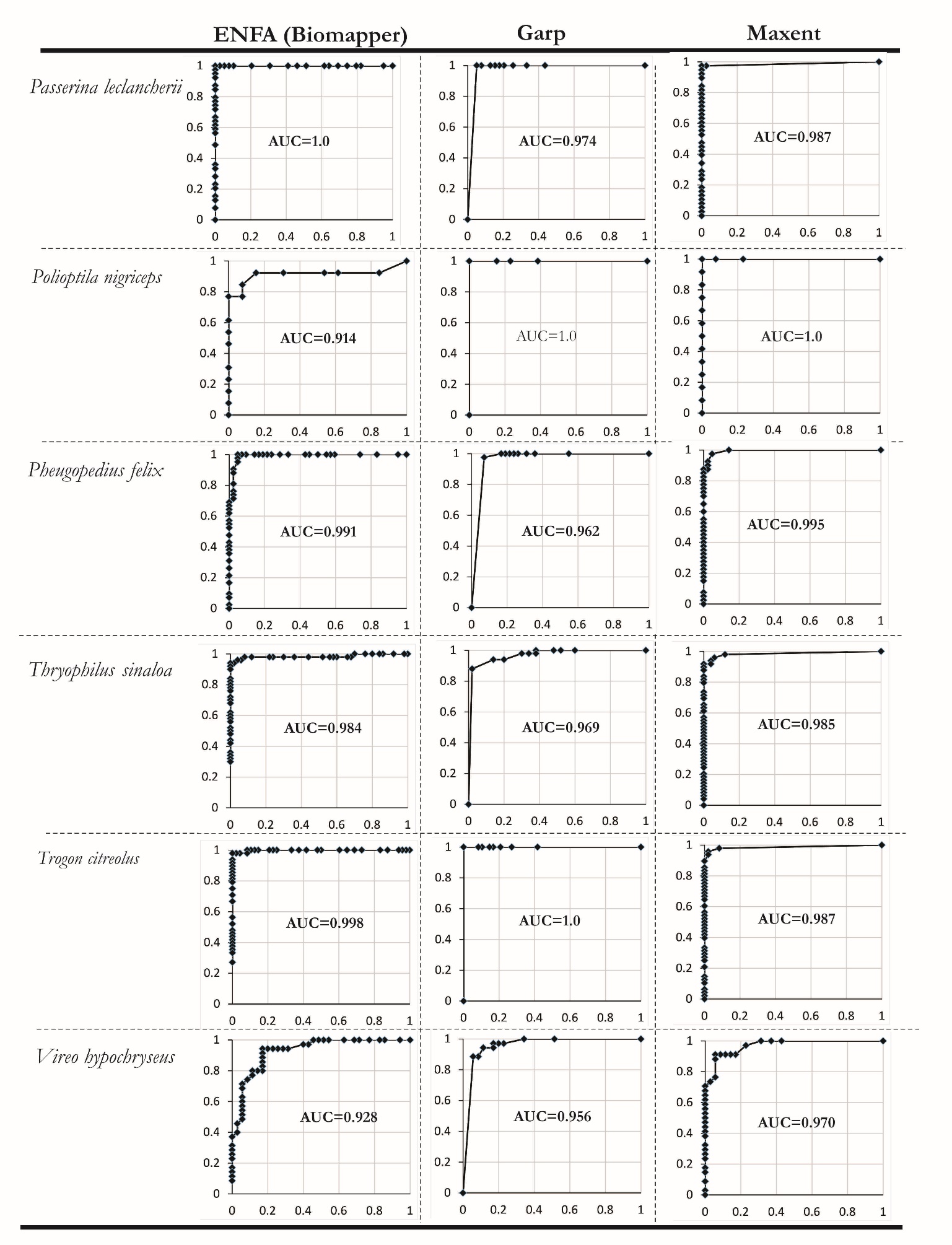 |
