# Supplementary material for: Validating distribution models for twelve endemic bird species of tropical dry forest in western Mexico
Source: Ecol Evol. 2017 Aug 19;7(19):7672–86. doi: 10.1002/ece3.3160 (PMC5632607; doi:10.1002/ece3.3160)
Supplement: Supplementary file 6 [file ECE3-7-7672-s006.docx]

| Appendix S6. Species potential distribution models (PDMs) for 12 endemic birds, obtained from applying three species distribution modeling algorithms (SDMA) (ENFA, Garp and Maxent). Models are displayed as continuous data stretched using two standard deviations (ENFA and Maxent; scale 0 to 100) and discrete data (Garp; scale 1 to 10). |
| --- |
|  |
| 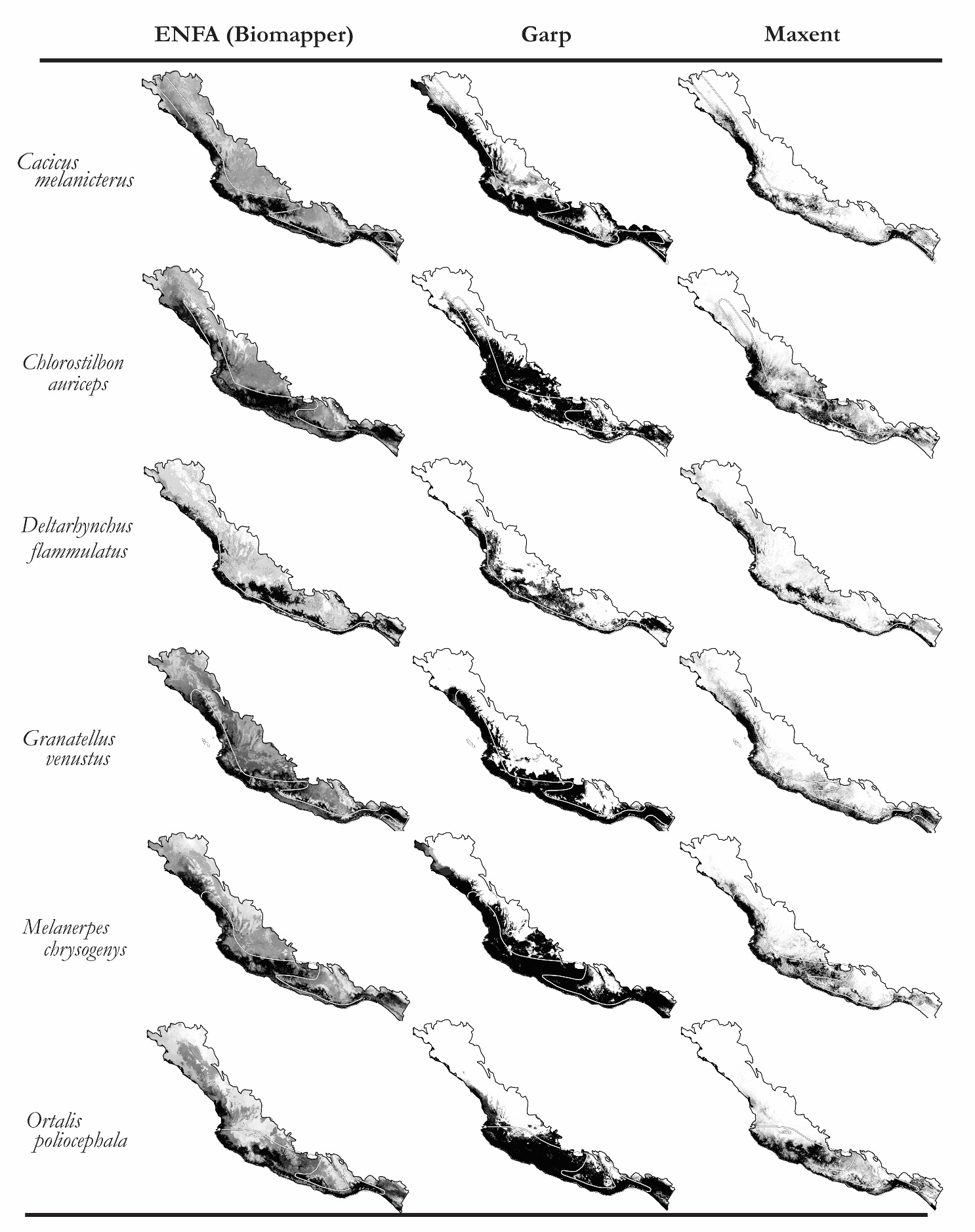 |

| Appendix S6 Cont. |
| --- |
|  |
| 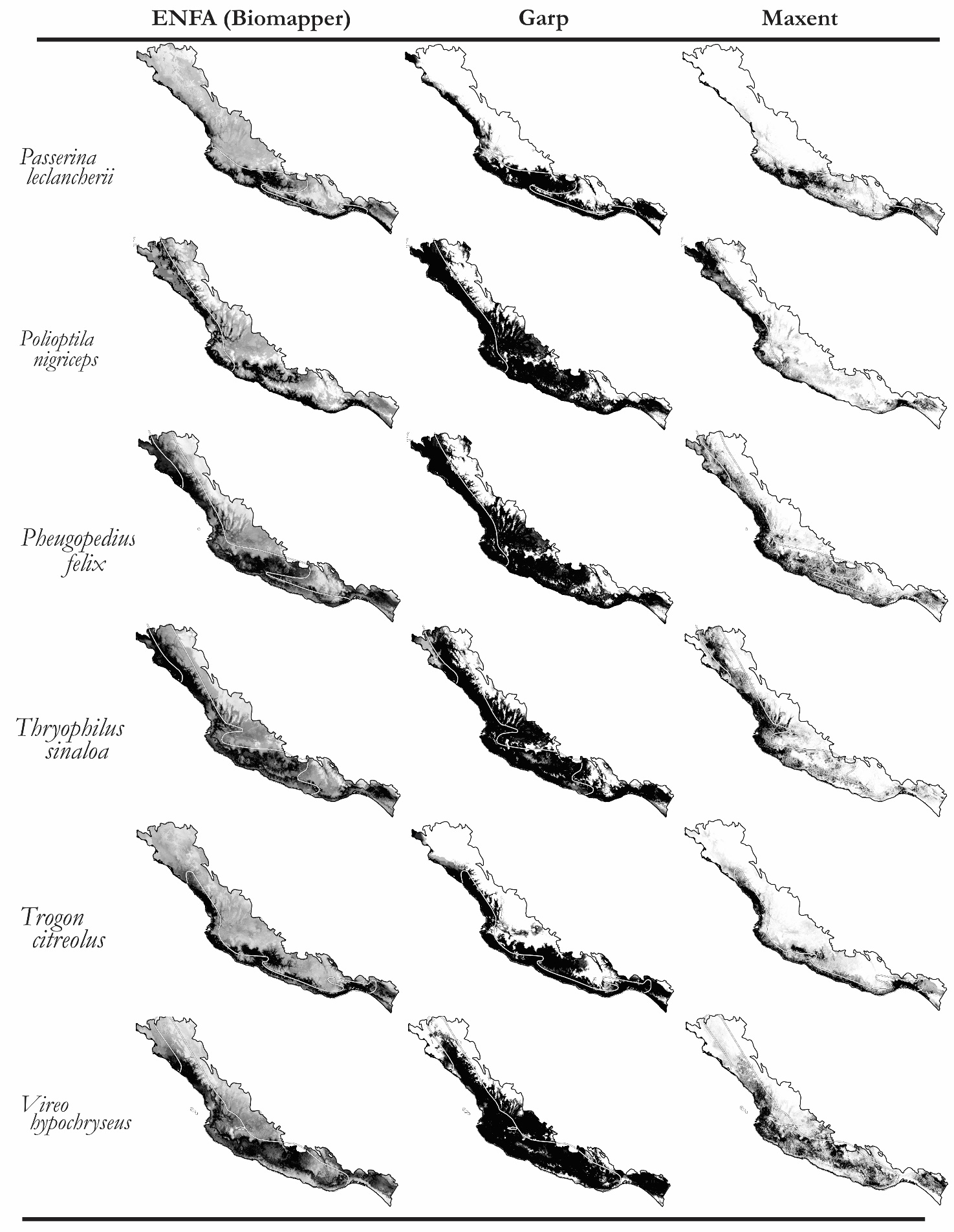 |
