# Supplementary material for: Validating distribution models for twelve endemic bird species of tropical dry forest in western Mexico
Source: Ecol Evol. 2017 Aug 19;7(19):7672–86. doi: 10.1002/ece3.3160 (PMC5632607; doi:10.1002/ece3.3160)
Supplement: Supplementary file 7 [file ECE3-7-7672-s007.docx]

| Appendix S7. Abbreviations used | |
| --- | --- |
| Abbreviation | Meaning |
| ADM | Species Actual Distribution Model |
| AUC | Area Under the Curve |
| ENFA | Ecological Niche Factor Analysis |
| FOD | Field species Occurrence Data |
| Garp | Genetic Algorithm for Rule Set Production |
| Maxent | Maximum Entropy |
| PDM | Species Potential Distribution Model |
| ROC | Receiver Operating Characteristic |
| SDM | Species Distribution Model |
| SDMA | Species Distribution Modeling Algorithm |
| SDMA | Species Distribution Model Algorithm |
| TDF | Tropical Dry Forest |
|  |  |
